# The prevalence of depression in patients with lumbar degenerative disk disease: A systematic review and meta-analysis

**DOI:** 10.1371/journal.pone.0322123

**Published:** 2025-05-07

**Authors:** Konstantin Li, Auyeskhan Dzhumabekov, Yermek Dyusembekov, Ardak Nurbakyt, Natalya Glushkova

**Affiliations:** 1 Department of Population Health and Social Sciences, Kazakhstan Medical University “KSPH”, Almaty, Kazakhstan; 2 Department of Clinical Work, Kazakhstan Medical University “KSPH”, Almaty, Kazakhstan; 3 Department of Neurosurgery, S.D. Asfendiyarov Kazakh National Medical University, Almaty, Kazakhstan; 4 Department of Public Health, S.D. Asfendiyarov National Medical University, Almaty, Kazakhstan; 5 Department of Epidemiology, Biostatistics, and Evidence-Based Medicine, Al-Farabi Kazakh National University, Almaty, Kazakhstan; Emory University School of Medicine, UNITED STATES OF AMERICA

## Abstract

**Background:**

Lumbar degenerative disc disease (DDD) is a prevalent musculoskeletal disorder characterized by significant pain, disability, and reduced quality of life. Depression frequently coexists with chronic pain conditions, intensifying symptoms and complicating management. Despite its clinical relevance, the prevalence of depression specifically among patients with lumbar DDD remains inadequately understood.

**Aim:**

This systematic review and meta-analysis aimed to assess the prevalence of depression among patients with lumbar DDD and to identify potential sources of heterogeneity.

**Methods:**

A comprehensive literature search was conducted using PubMed, Web of Science, ScienceDirect, Academic Search Complete, and Google Scholar to identify relevant studies published between 2015 and 2022. Studies reporting the prevalence of depression among patients with lumbar DDD were included in the analysis. Pooled prevalence estimates were calculated using random-effects models, and subgroup analyses were performed to investigate sources of heterogeneity.

**Results:**

Seven studies met the inclusion criteria, encompassing 3485 patients with lumbar DDD. The pooled prevalence of depression was estimated at 20.19% (95% CI 8.94–39.46%). Subgroup analyses revealed significant variations in prevalence estimates across different geographic regions and assessment tools. Sensitivity analyses confirmed the robustness of the findings, while meta-regression analyses did not identify significant associations between prevalence rates and factors such as age or year of publication.

**Conclusions:**

This study highlights a substantial burden of depression among patients with lumbar DDD, emphasizing the need for comprehensive mental health assessment and management in this population. The findings contribute to a better understanding of the psychological comorbidities associated with lumbar DDD and have implications for clinical practice and future research.

## Introduction

The intervertebral disc undergoes biochemical changes and dehydration as part of the normal aging process [1]. Additionally, both macro and micro traumas contribute to the natural degeneration of the disc over time [1]. The lumbar spine’s susceptibility to degeneration may be attributed to the evolutionary transition to bipedalism and an upright posture [2]. Lumbar degenerative disk disease (DDD) affects both sexes equally and does not always present with symptoms [3,4]. However, by the age of 50, more than 85% of the population exhibits changes associated with disc degeneration [4]. DDD represents a significant health burden, contributing substantially to back pain and radiculopathy, which can lead to disability, decreased quality of life, and increased healthcare costs [5,6]. Coexisting with these painful conditions, depression has been identified as a prevalent comorbidity [7].

Depression, recognized as a global health priority, constitutes a substantial portion of the global non-fatal disease burden[8]. Evidence indicates that depression is associated with increased pain, greater disability, and prolonged recovery in patients with chronic lower back pain [9,10]. Moreover, understanding the intricate relationship between physical and psychological factors in lumbar DDD patients is essential for optimizing treatment outcomes and enhancing overall well-being. Research suggests that depression can exacerbate pain perception, reduce functional capacity, and hinder responsiveness to conventional treatments [11]. Conversely, effective management of depressive symptoms has been associated with improved pain control, functional status, and overall treatment satisfaction in chronic pain populations [12]. Therefore, elucidating the prevalence of depression among lumbar DDD patients not only sheds light on the extent of the mental health burden in this population but also underscores the importance of integrated, multidisciplinary approaches to patient care. By addressing both the physical and psychological aspects of the condition, healthcare providers can implement more holistic and effective management strategies, ultimately improving patient outcomes and quality of life [13]. A clear understanding of the prevalence of depression among lumbar DDD patients is, therefore, essential for ensuring comprehensive and targeted disease management.

Although a meta-analysis of published literature reports a pooled prevalence rate of depression among degenerative spine disease patients at 30.8%, substantial variations exist due to differences in assessment methods, sample sizes, and patient populations [7]. Furthermore, no comprehensive meta-analysis has systematically examined the prevalence of depression specifically in patients with lumbar DDD.

The primary objective of this study was to investigate the prevalence of depression among patients with lumbar DDD through a systematic review of the published literature. Additionally, the study aimed to identify potential sources of heterogeneity using subgroup analysis, meta-regression analysis, and sensitivity analysis. This study aims to offer a more comprehensive understanding of depression prevalence among lumbar DDD patients, which is crucial for guiding interventions to prevent and manage mental health disorders in this population.

## Materials and methods

The study protocol is registered with the PROSPERO International Prospective Register of Systematic Reviews CRD42024489684.

### Search strategy

The PROSPERO database was searched to identify registrations of similar studies, but no were found. Subsequently, a search was conducted in five major electronic literature databases: PubMed, Web of Science, ScienceDirect, Academic Search Complete, and Google Scholar. The literature search in the specified sources was initiated on January 15, 2023, and completed on March 7, 2024. The search strategy included the following keywords: “depression”; “depressive disorder”; “degenerative disk disease”; “degenerative disk”; and “prevalence”. The full strategy is presented in the supplementary materials (S1 Table).

### Eligibility criteria

Methodologically, the literature screening and synthesis followed the recommendations of Preferred Reporting Items for Systematic Reviews and Meta-Analyses (PRISMA). Two authors (K.L. & Y.K.D.) independently performed the literature screening. The inclusion criteria for the studies: (a) full-text publications in peer-reviewed journals; (b) studies reporting data on patients with lumbar degenerative disc disease (DDD); (c) studies reporting depression identified through a validated self-report questionnaire. The exclusion criteria for the studies: (a) studies evaluating the psychometric properties of instruments; (b) studies including all patients with spinal degenerative diseases or presenting data on patients undergoing certain types of surgeries without specifying the population; (c) studies with duplicative data; (d) studies reporting only the number of patients with clinically diagnosed depression or those receiving antidepressant treatment; (e) studies reporting mean scores on the psychometric questionnaire but not the prevalence of depression among DDD patients.

### Selection of studies and data extraction

The identified publications underwent deduplication followed by primary (title + abstract) and eligibility (full text) reviews. Subsequently, full-text examination, review, and data extraction were performed, with each step resulting to the exclusion of publications based on predefined inclusion and exclusion criteria. Following the PRISMA guidelines, two authors (K.L. & Y.K.D.) independently extracted the following information from the identified full-text articles using a standardized data extraction form. The data of interest included: 1) first author’s name, 2) publication year, 3) country, 4) study design, 5) sample size, 6) lesion location, 7) number of females, 8) number of smokers, 9) mean age, 10) type of work (physical, intellectual), 11) depression prevalence, 12) Visual Analogue Scale (VAS) scores, 13) assessment method and criteria, and 14) Oswestry Disability Index (ODI) scores. Any disagreements between the authors were resolved through consensus, with a discussion involving the third author (N.G.).

### Risk of bias

The Critical Appraisal Skills Programme (CASP) Qualitative Research Checklist was used to evaluate the methodological quality of the included studies [14]. This checklist comprised ten questions covering various aspects, including the study’s objectives, methodology, research design, recruitment approach, data collection methods, researcher-participant relationships, ethical considerations, data analysis, research findings, and overall value. Each criterion was assessed with a rating of ‘yes’ when adequately described (scored as 1), ‘no’ when absent (scored as 0), and ‘can’t tell’ when unclear or incomplete (scored as 0.5). Total scores ranged from 0 to 10, with a score of at least 7 indicating satisfactory quality.

### Statistical analysis

The pooled mean depression prevalence with 95% confidence intervals (95% CI) was calculated using random-effects model for meta-analysis in RStudio software [15]. Forest plots were used to display the pooled estimates. Heterogeneity across studies was assessed using the I²-statistic. Sensitivity analysis was conducted to investigate which study significantly influences the pooled prevalence estimates. Additionally, meta-regression analyses were performed for age and baseline survey year. Subgroup analysis was carried out to explore sources of heterogeneity, stratifying studies by region, ODI scores, VAS scores, and assessment method. Publication bias was evaluated through visual inspection of a drapery plot and statistical analysis using Egger’s test, examining potential asymmetry in the distribution of study results.

## Results

**Fig 1:**
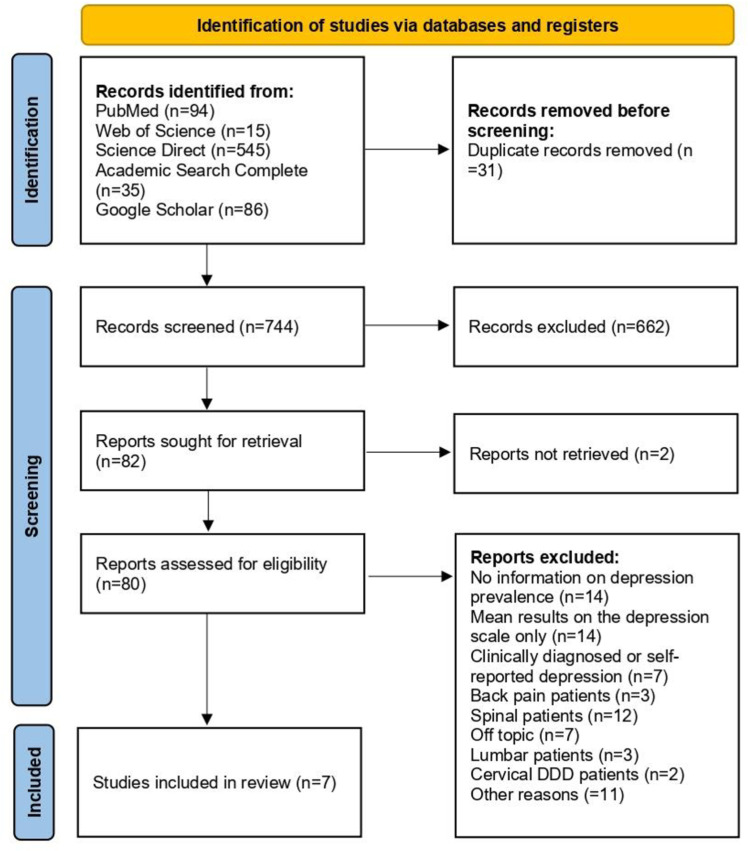
PRISMA flow chart of study selection.

A thorough search across PubMed, Web of Science, ScienceDirect, Academic Search Complete, and Google Scholar databases yielded 775 records. Initial screening reduced this number to 744 non-duplicative records, from which 80 full-text articles underwent evaluation. Among these studies, 13 presented mean depression scale scores without providing information on the number of patients involved [16–28], and six studies presented the prevalence of clinically diagnosed depression [29–34]. Ultimately, seven articles met the inclusion criteria for the systematic review. The study selection process is illustrated in Fig 1 [35]. A detailed table of the study selection process is presented in Supplementary S2 Table.

### Description of included studies and subjects

The study designs and characteristics of patients are presented in Table 1. All the studies were published between 2015 and 2022. Four studies were conducted in Europe, two in the Middle East, and one in Asia. Most of the studies were prospective, with only one being retrospective. A total of 3485 patients with lumbar DDD were included in seven studies (mean sample size = 497.86 patients, range = 51–1505 patients). The mean age of the participants ranged from 42.7 to 58.3 years. The proportion of female participants ranged from 43% to 70%. Three studies did not report ODI and VAS scores. Four studies used the Beck Depression Inventory (BDI), two studies used the Zung Self-Rating Depression Scale (ZDS), and one study used the 12-Item Short Form Survey (SF-12). A detailed data extraction table is provided in Supplementary S3 Table.

**Table 1:** Summary of included articles.

First author, year	Country	Region	Study design	Patient number	Age (mean±SD)	Female (%)	ODI (mean±SD)	VAS (mean±SD)	Depression cut-off
Wang 2015 [36]	China	Asia	prospective	749	58.3 ± 10.3	437 (58%)	67.3 ± 5.7	6.59 ± 0.62	ZDS ≥ 60
Engel-Yeger 2016 [37]	Israel	Middle East	prospective	51	46.96 ± 14.36	27 (53%)	40.79 ± 15.71	6.02 ± 2.21	BDI ≥ 17
Jablonska 2017 [38]	Poland	Europe	prospective	140	42.7 ± 10.99	98 (70%)	not reported	not reported	BDI (group II-IV)
Stienen 2017 [39]	Switzerland	Europe	prospective	375	55.0 + -15.3	162 (43%)	56.19 ± 17.48	4.33 ± 2.81	SF-12 (Q1-Q2)
Telli 2020 [40]	Turkey	Middle East	prospective	234	46.17 ± 11.61	148 (63%)	not reported	not reported	BDI ≥ 14
Biczo 2022 [41]	Hungary	Europe	prospective	431	52.7 ± 13.9	264 (61%)	47.4 ± 18.4	7.2 ± 1.9	ZDS ZDS ≥ 50
Mertimo 2022 [42]	Finland	Europe	retrospective	1505	47 ± 0.4	799 (53%)	not reported	not reported	BDI ≥ 13

Abbreviations: BDI: Beck Depression Inventory; ODI: The Oswestry Disability Index; SD: Standard deviation; SF-12: 12-Item Short Form Survey; VAS: The Visual Analogue Scale; ZDS: Zung Self Rating Depression Scale;

### The risk of bias assessment

The risk of bias assessment results are presented in Table 2. All the studies had low risk of bias with a CASP score above 8.5.

**Table 2:** Risk of bias assessment results.

Author, year	Aim	Methodology	Design	Recruitment	Data collection	Relationship	Ethical	Data analysis	Finding	Values	Score
Wang 2015 [36]	Yes	Yes	Yes	Yes	Yes	No	Yes	Yes	Yes	Yes	9
Engel-Yeger 2016 [37]	Yes	Yes	Yes	Yes	Can’t tell	No	Yes	Yes	Yes	Yes	8.5
Jablonska 2017 [38]	Yes	Yes	Yes	Yes	Yes	No	Yes	Yes	Yes	Yes	9
Stienen 2017 [39]	Yes	Yes	Yes	Yes	Yes	No	Yes	Yes	Yes	Yes	9
Telli 2020 [40]	Yes	Yes	Yes	Yes	Yes	No	Yes	Yes	Yes	Yes	9
Biczo 2022 [41]	Yes	Yes	Yes	Yes	Yes	No	Yes	Yes	Can’t tell	Yes	8.5
Mertimo 2022 [42]	Yes	Yes	Yes	Yes	Yes	Yes	Yes	Yes	Yes	Yes	9

### Prevalence rate

**Fig 2:**
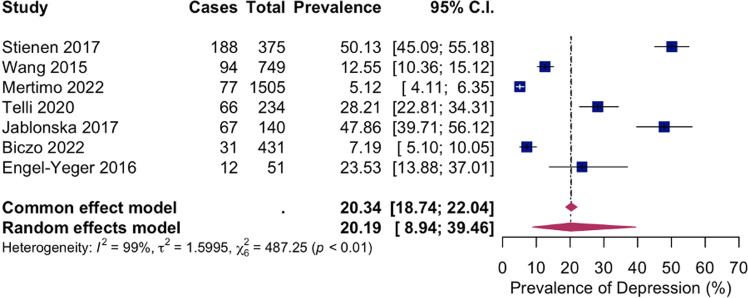
Meta-analysis of the depression prevalence in lumbar DDD patients. Abbreviations: C.I.: confidence interval.

Based on the random-effects model, the pooled prevalence estimate of seven studies on depression in lumbar DDD patients, either before operative treatment or on conservative therapy, was 20.19% (535/3485 participants; [95% CI 8.94–39.46%]). The test for heterogeneity indicated high heterogeneity: I2 = 99%, Q (df = 6)=487.25, p-value<0.001 (Fig 2).

### Sensitivity analysis

**Fig 3:**
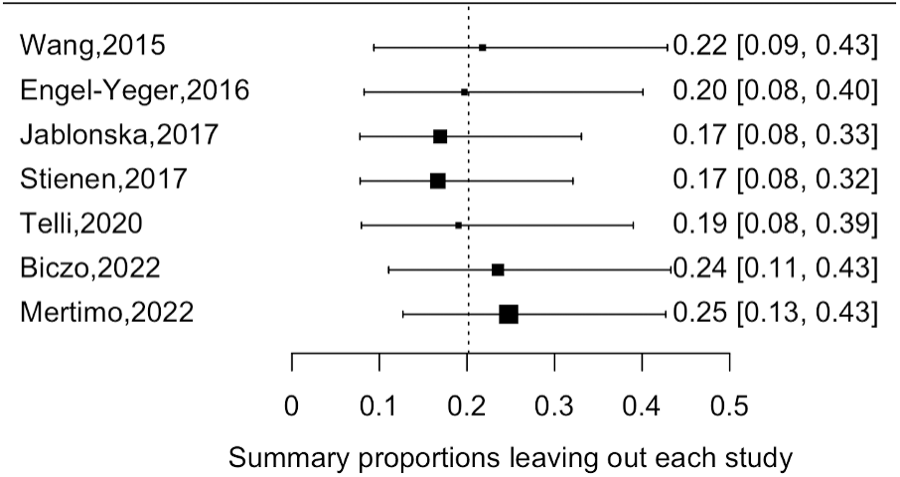
Leave-one-out analysis results.

Sequential exclusion of individual studies in a sensitivity analysis assessed the robustness of the pooled estimate. The results revealed persistent heterogeneity, but the pooled prevalence estimate remained stable (S3 Fig). Additionally, the leave-one-out study analysis identified the Mertimo 2022 study as the most influential, with its results presented in the supplemental materials (Fig 3).

### Meta-regression results

Random-effects multivariate meta-regressions were performed to explore the source of heterogeneity. Meta-regression analyses based on age and year of publication did not demonstrate a statistically significant association (p values for the model of 0.64 and 0.12, respectively). This is likely due to low power and the small number of available studies and should not be interpreted as evidence of no effect of age or year on depression prevalence. Meta-regression results are available in the supplemental materials (S1 Fig and S2 Fig).

### Subgroup analysis

To explore potential sources of heterogeneity, subgroup analyses were conducted in each subgroup. Statistically significant differences in prevalence estimates were detected with high heterogeneity across the studies, including comparisons between regions (Asia vs. Middle East vs. Europe): (12.55% [95% CI 10.36–15.12%] vs 25.85% [95% CI 4.90–70.21%] vs 19.97% [95% CI 6.15–48.70%] I^2^ = 99.33, p < 0.01). Other subgroup analyses included comparisons based on ODI scores: ODI ≥ 50 vs. ODI score <50 vs. ODI score not reported 27.54% [95% CI 5.69–70.54%] I^2^ = 99.40 vs 13.21% [95% CI 2.29–49.74%] I^2^ = 99.43 vs 21.12% [95% CI 5.60–54.73%] I^2^ = 99.12, p < 0.01); studies with VAS scores: VAS ≥ 6 vs. VAS score <6 vs. VAS score not reported 12.95% [95% CI 3.83–35.70%] I^2^ = 86.93 vs 50.13% [95% CI 7.65–72.25%] vs 21.10% [95% CI 6.87–49.24%] I^2^ = 99.12, p < 0.01); and studies using ZDS vs. BDI vs. SF-12 9.56% [95% CI 2.10–34.29%] I^2^ = 87.63 vs 20.66% [95% CI 8.15–46.29%] I^2^ = 98.69 vs 50.13% [95% CI 45.09–55.18%], p < 0.01) (Table 3). Additional visualization of the results is presented in the supplemental materials (S4–S7 Figs).

**Table 3:** Subgroup analysis of the meta-analysis of the depression prevalence in lumbar DDD patients.

	Depression prevalence (%)	95% CI	I^2^ (%)	95% CI	p _subgroup_
**Region**					<0.01
Asia	12.55	10.36-15.12	–	–	
Middle East	25.85	4.90-70.21	–	–	
Europe	19.97	6.15-48.70	99.33	99.04-99.53	
**ODI score**					<0.01
≥50	27.54	5.69-70.54	99.40	98.92-99.66	
<50	13.21	2.29-49.74	92.43	74.35-97.77	
not reported	21.12	5.60-54.73	99.12	98.59-99.45	
**VAS score**					<0.01
≥6	12.95	3.83-35.70	86.93	62.67-95.42	
<6	50.13	7.65-72.25	–	–	
not reported	21.10	6.87-49.24	99.12	98.59-99.45	
**Scale**					<0.01
ZDS	9.56	2.10-34.29	87.63	52.05-96.81	
BDI	20.66	8.15-46.29	98.69	97.96-99.16	
SF-12	50.13	45.09-55.18	–	–	

Abbreviations: BDI: Beck Depression Inventory; CI: confidence interval; ODI: The Oswestry Disability Index; SF-12: 12-Item Short Form Survey; VAS: The Visual Analogue Scale; ZDS: Zung Self Rating Depression Scale;

### Publication bias assessment

**Fig 4:**
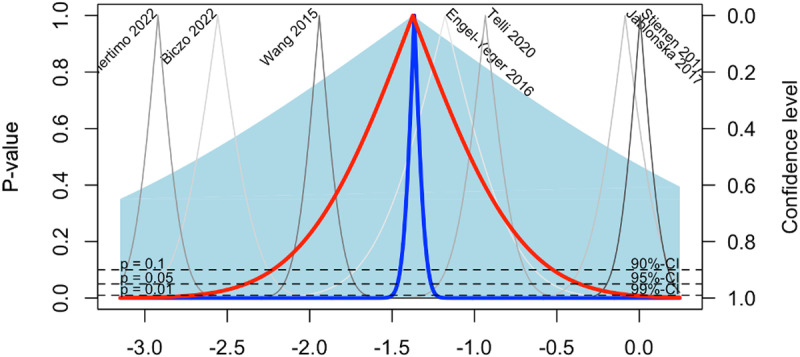
Drapery plot assessing publication bias.

Upon visual inspection of the drapery plot (Fig 4), no evident asymmetry was observed, suggesting a relatively symmetric distribution of study results around the estimated effect size. This finding was further confirmed by Egger’s test for publication bias, which yielded non-significant results (p = 0.93). The absence of asymmetry in the drapery plot, coupled with the non-significant Egger’s test, suggests minimal evidence of publication bias in the meta-analysis of lumbar DDD studies. These findings enhance confidence in the robustness and validity of the meta-analytic results.

## Discussion

The meta-analysis revealed a significant pooled prevalence of depression among lumbar DDD patients, whether preoperatively or during conservative treatment, at 20.19%. This finding highlights a notable burden of depressive symptoms within this demographic. Recognizing the potential benefits of preoperative depression management in alleviating postoperative pain intensity, recurrence, and opioid misuse [29,33], underscores the critical need to scrutinize the high incidence of preoperative depression diagnosis. Consequently, there is an imperative for integrating comprehensive psychological assessments and interventions into the preoperative care regimen. Such an approach is pivotal in bolstering both physical and mental health outcomes among lumbar DDD patients.

The effectiveness of antidepressants in managing patients with lower back pain and DDD further supports our findings on the high prevalence of depression among lumbar DDD patients. A recent systematic review of clinical practice guidelines indicates that antidepressants are the most recommended class of medications among the eleven guidelines analyzed [43]. Additionally, a meta-analysis of nine randomized clinical trials confirms the efficacy of duloxetine in managing nonspecific chronic back pain, not only in pain relief but also in improving quality of life [44]. While surgical treatment of lumbar DDD significantly reduces pain at three months post-operatively, its effect on depression symptoms is limited [45]. Postoperative outcome measures after lumbar spine surgery indicate that patients with depression exhibit worse disease severity both before and after surgery; however, they also show a higher potential for improvement in disability, pain, and physical function [46].

Our study findings underscore the importance of critically defining the study population when interpreting results in comparison with similar studies. Notably, Chen et al. (2021) reported a substantial prevalence of depressive symptoms among individuals with degenerative spine disease at 30.8% (7). However, our study found a lower prevalence rate, possibly due to the specificity of focusing solely on lumbar DDD patients, a smaller sample size, and the use of higher cut-off scores for depression diagnosis used in the included studies. The exclusion of patients with cervical DDD was based on well-established differences, not only in disease location but also in symptomatology, functional impact, complications, treatment modalities, and potentially, depression prevalence when compared to lumbar DDD patients [47,48].

Acknowledging the regional focus of the included studies, primarily on European and Middle Eastern populations, is crucial, as it may limit the generalizability of findings to other geographic regions. The lack of postoperative assessment data in our study prevents the evaluation of depressive symptom progression following operative treatment. Additionally, our exclusion criteria aimed to reduce heterogeneity by excluding patients with clinically diagnosed depression, those on antidepressant treatment, or those self-reporting as depressed, focusing solely on self-report questionnaire results.

Despite efforts to reduce bias, variability in depression assessment tools and cutoff scores across studies may have influenced the pooled prevalence estimate and introduced heterogeneity. This variability underscores the need for standardization in assessment protocols to enhance the comparability of findings across studies.

In conclusion, our study provides valuable insights into the prevalence of depression among lumbar DDD patients. The high prevalence of depression underscores the necessity for comprehensive psychological assessment and management in this population, including the use of antidepressants when appropriate. Future research should address identified limitations, such as the need for more diverse study populations, postoperative assessment data, and standardized assessment protocols, to inform targeted interventions that address both physical and mental health outcomes in this population.

## Supporting information

S1 Table The full search strategy. (DOCX)

S2 Table Study selection process results. (XLSX)

S3 Table Comprehensive data extraction details. (XLSX)

S1 Fig Meta-regression analysis based on the mean age of participants. (TIF)

S2 Fig Meta-regression analysis based on the year of publication. (TIF)

S3 Fig Influential study analysis. (TIF)

S4 Fig Subgroup analysis of depression prevalence in lumbar DDD patients by region. (TIF)

S5 Fig Subgroup analysis of depression prevalence in lumbar DDD patients by ODI score category. (TIF)

S6 Fig Subgroup analysis of depression prevalence in lumbar DDD patients by VAS score category. (TIF)

S7 Fig Subgroup analysis of depression prevalence in lumbar DDD patients by assessment scale. (TIF)

S1 File PRISMA 2020 checklist for systematic review. (DOCX)

S2 File Prospero protocol. (PDF)
