# Supplementary material for: The prevalence of depression in patients with lumbar degenerative disk disease: A systematic review and meta-analysis
Source: PLoS One. 2025 May 7;20(5):e0322123. doi: 10.1371/journal.pone.0322123 (PMC12057953; doi:10.1371/journal.pone.0322123)
Supplement: S1 Table — (DOCX) [file pone.0322123.s001.docx]

| 1. degenerative disc disease [Title/Abstract] 2. degenerative disk [Title/Abstract] 3. OR/1-2 4. "Depression"[Mesh] 5. "Depressive Disorder"[Mesh] 6. "Depressive Disorder, Major"[Mesh] 7. depression [Title/Abstract] 8. depressive disorder [Title/Abstract] 9. major depressive disorder [Title/Abstract] 10. major depression [Title/Abstract] 11. MDD[Title/Abstract] 12. depressed [Title/Abstract] 13. sadness [Title/Abstract] 14. distress*[Title/Abstract] 15. suicide [Title/Abstract] 16. OR/4-15 17. "Prevalence"[Mesh] 18. "Epidemiologic Studies"[Mesh] 19. "Epidemiology"[Mesh:NoExp] 20. prevalence*[Title/Abstract] 21. epidemiolog*[Title/Abstract] 22. OR/17-21      1. 3 AND 16 AND 22 |
| --- |
