# Supplementary figures and images for: The prevalence of depression in patients with lumbar degenerative disk disease: A systematic review and meta-analysis
Source: PLoS One. 2025 May 7;20(5):e0322123. doi: 10.1371/journal.pone.0322123 (PMC12057953; doi:10.1371/journal.pone.0322123)

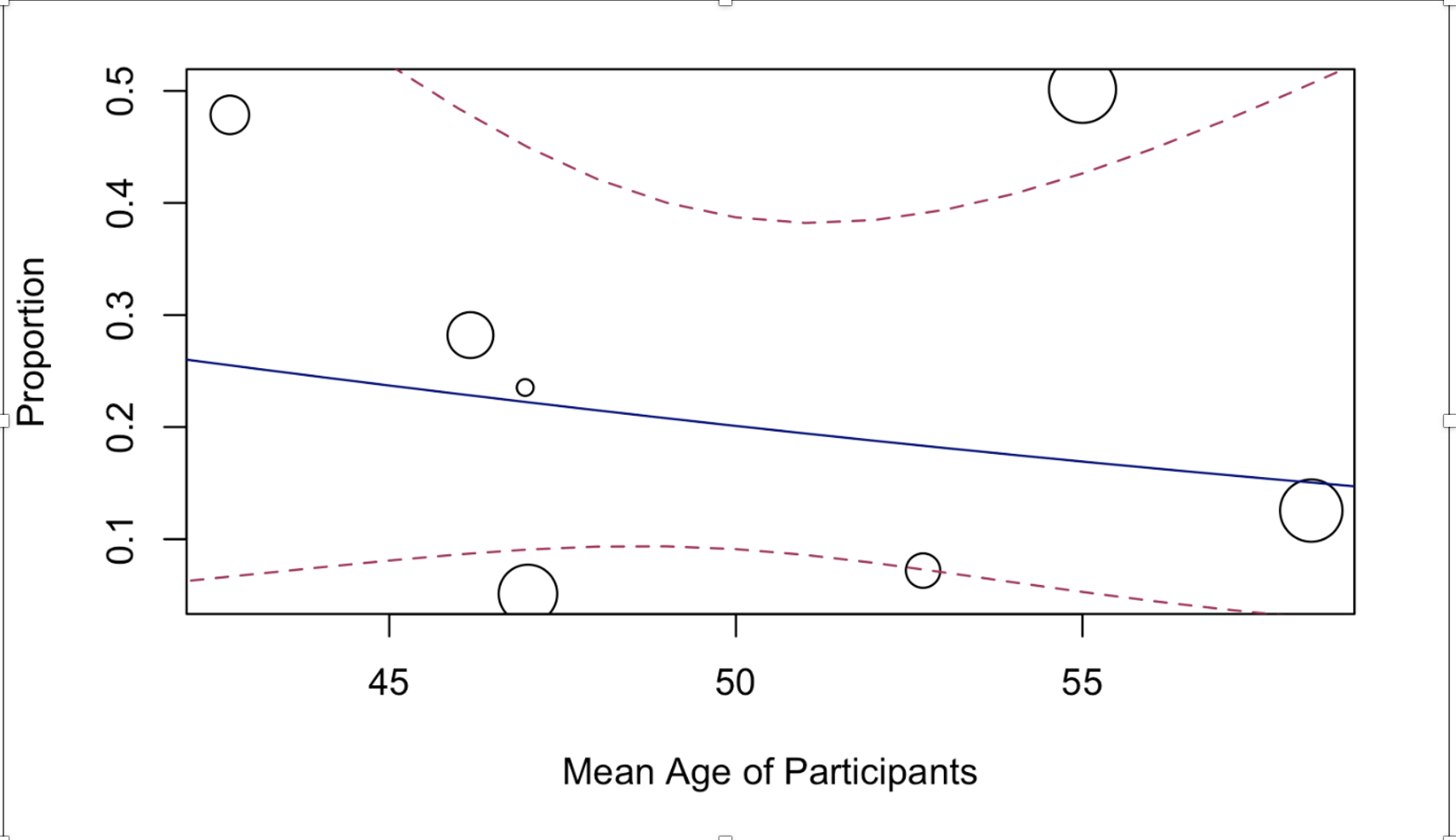

Supplement: S1 Fig — (TIF) [file pone.0322123.s002.tif]

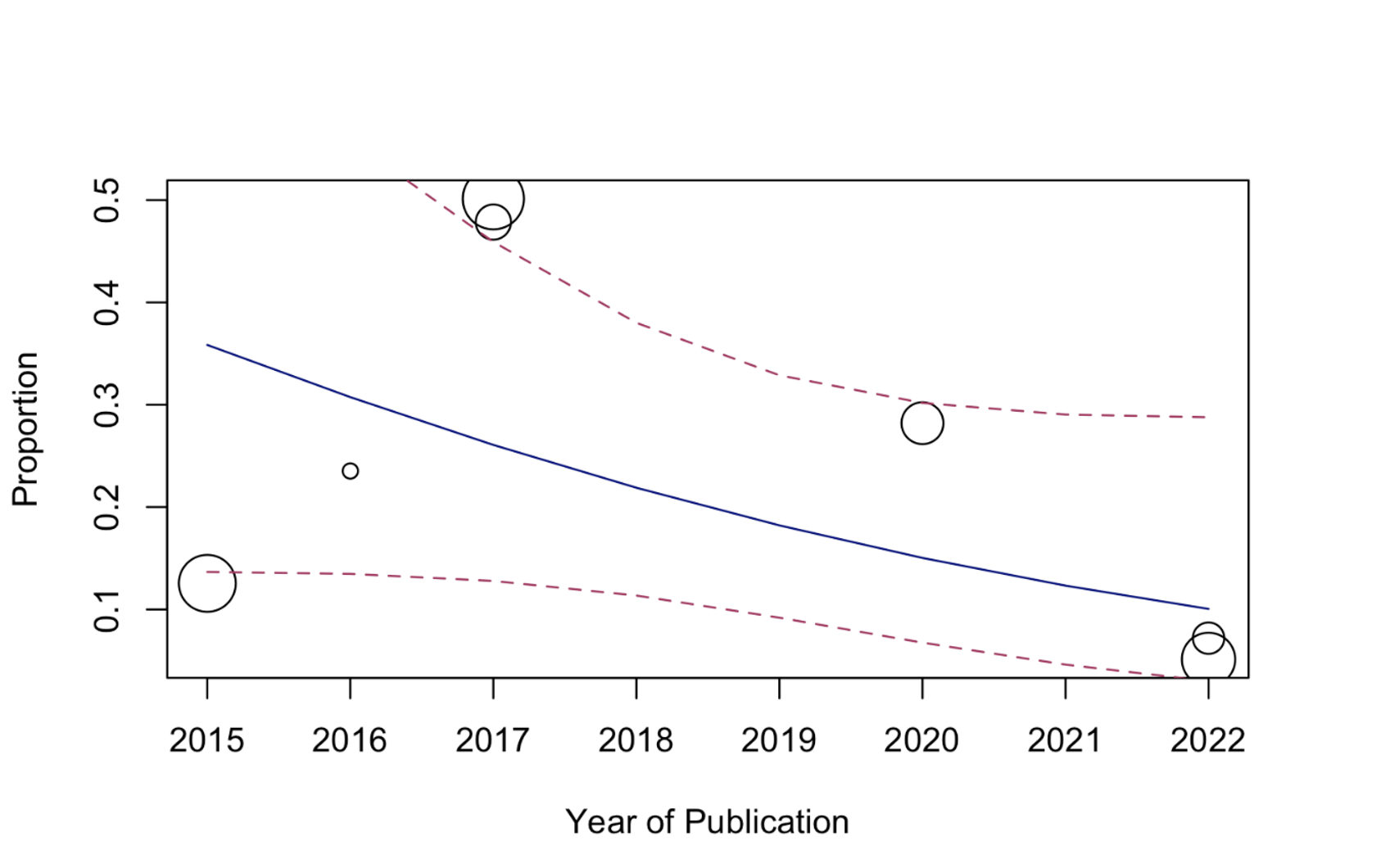

Supplement: S2 Fig — (TIF) [file pone.0322123.s003.tif]

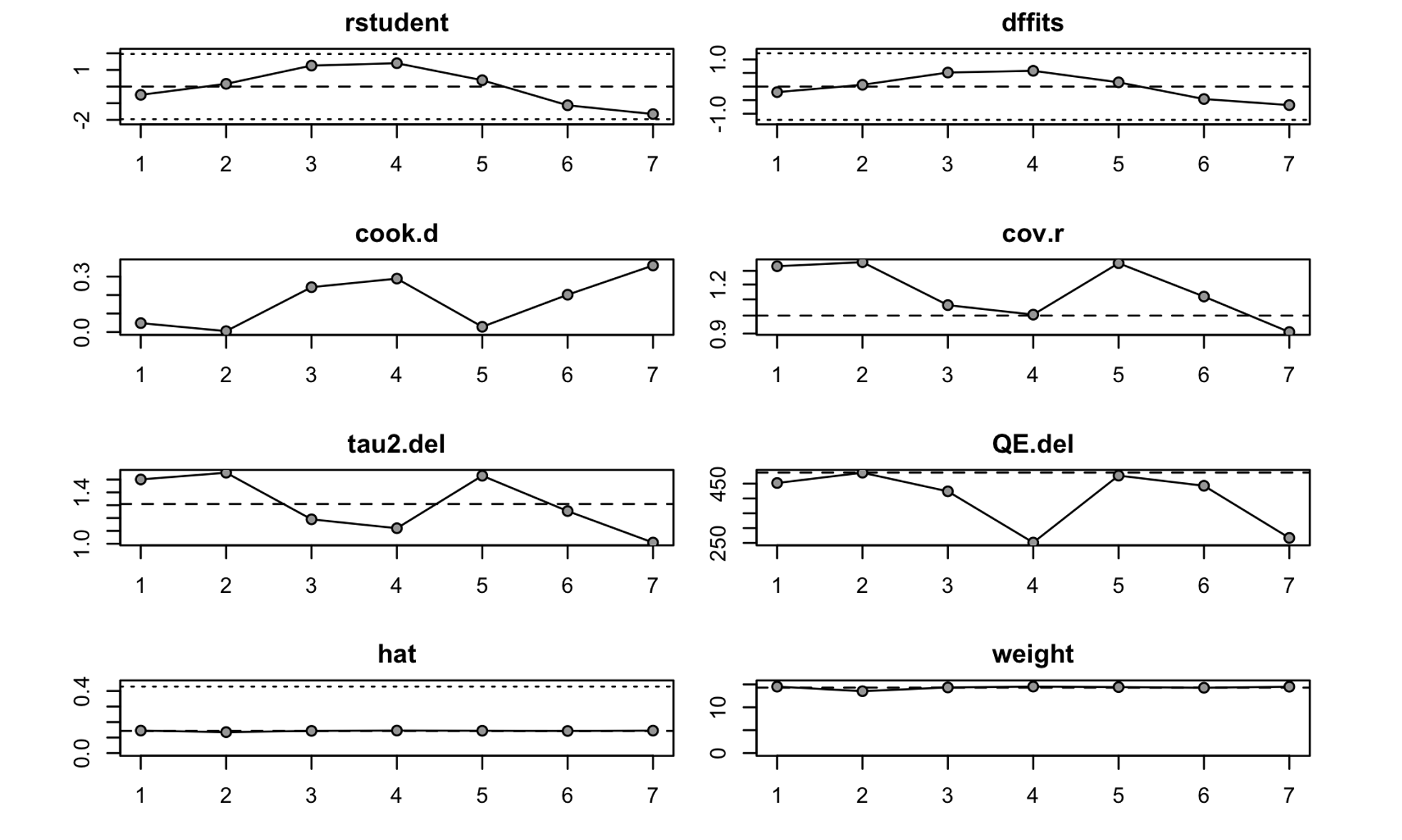

Supplement: S3 Fig — (TIF) [file pone.0322123.s004.tif]

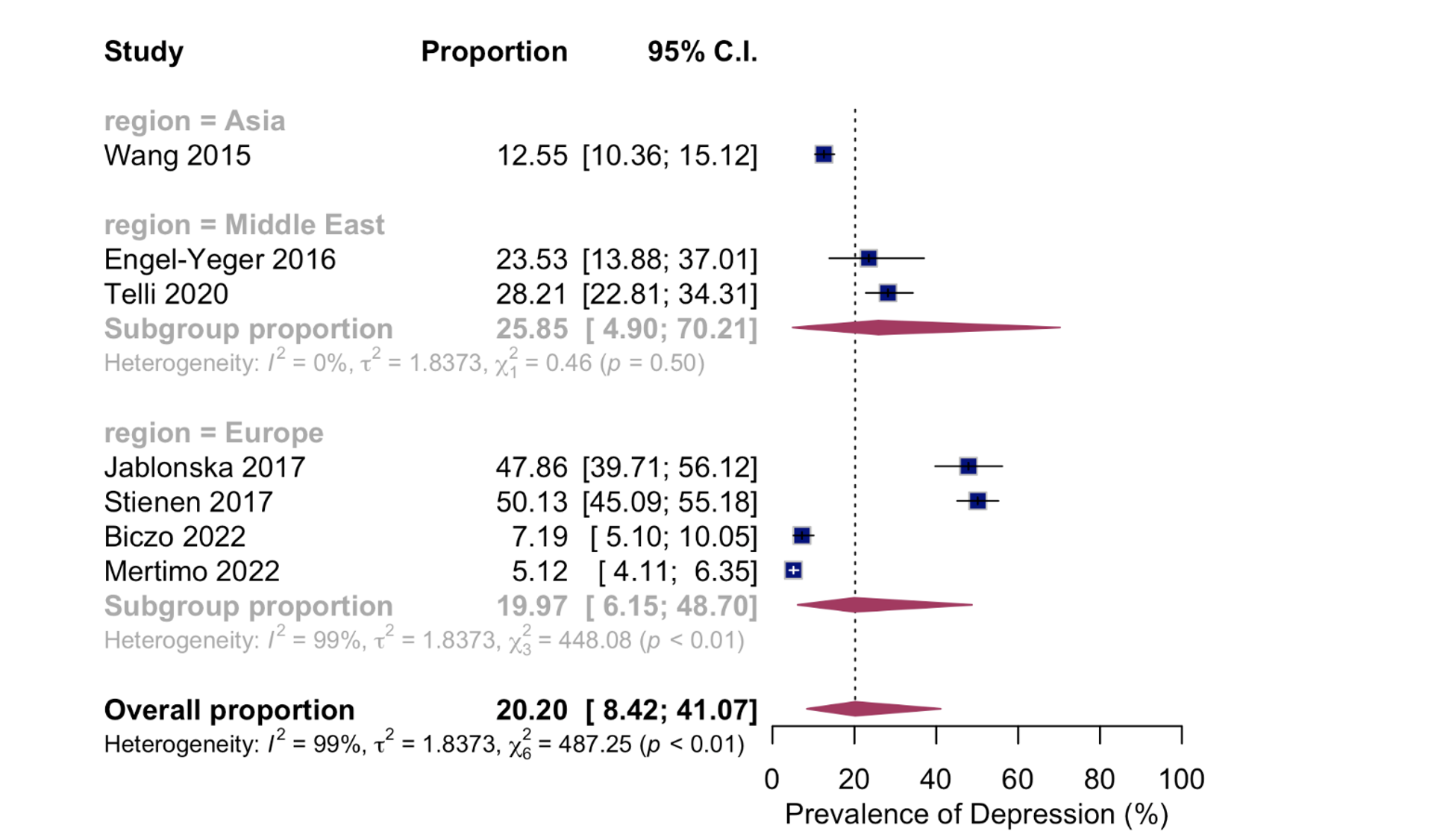

Supplement: S4 Fig — (TIF) [file pone.0322123.s005.tif]

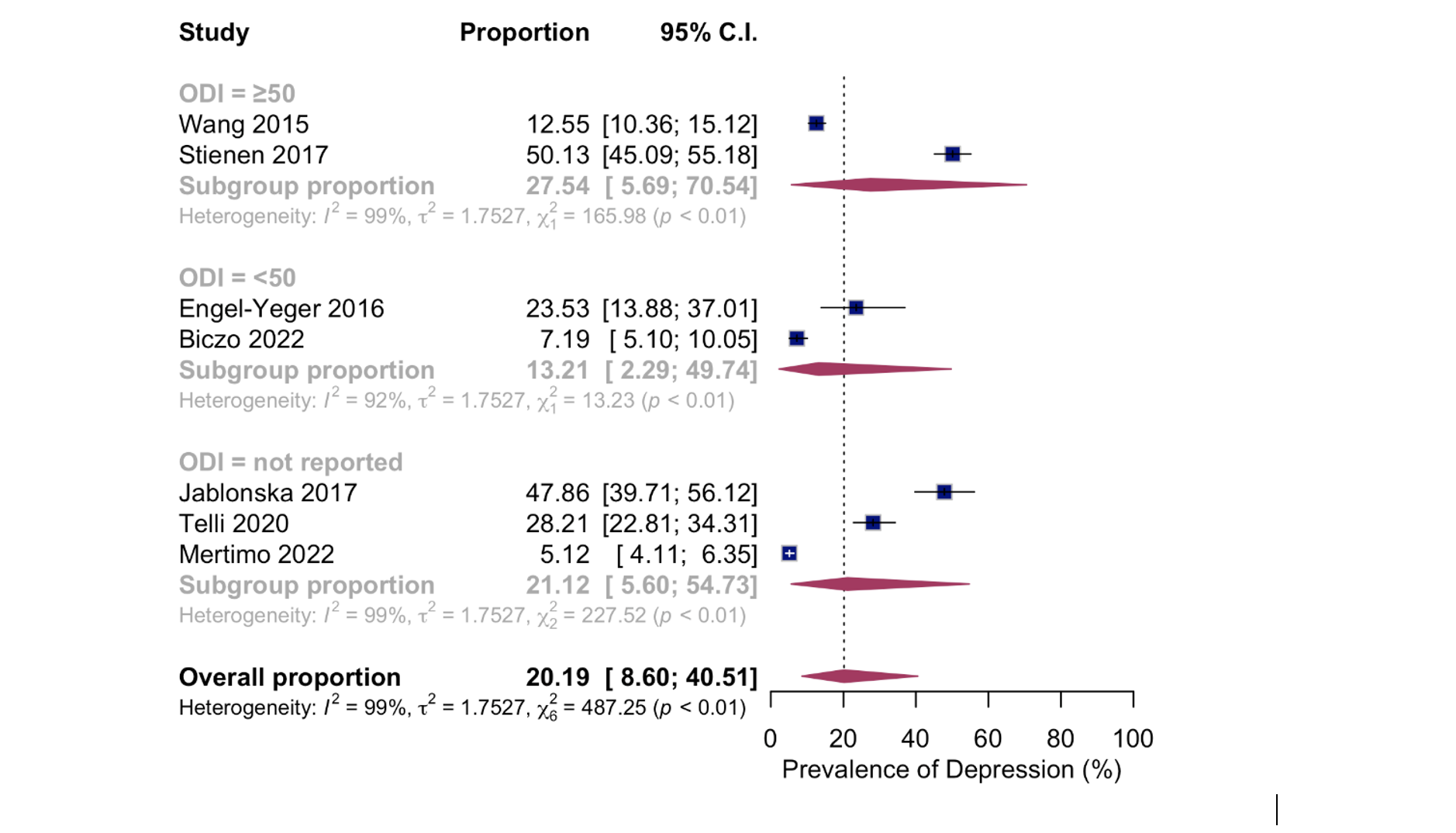

Supplement: S5 Fig — (TIF) [file pone.0322123.s006.tif]

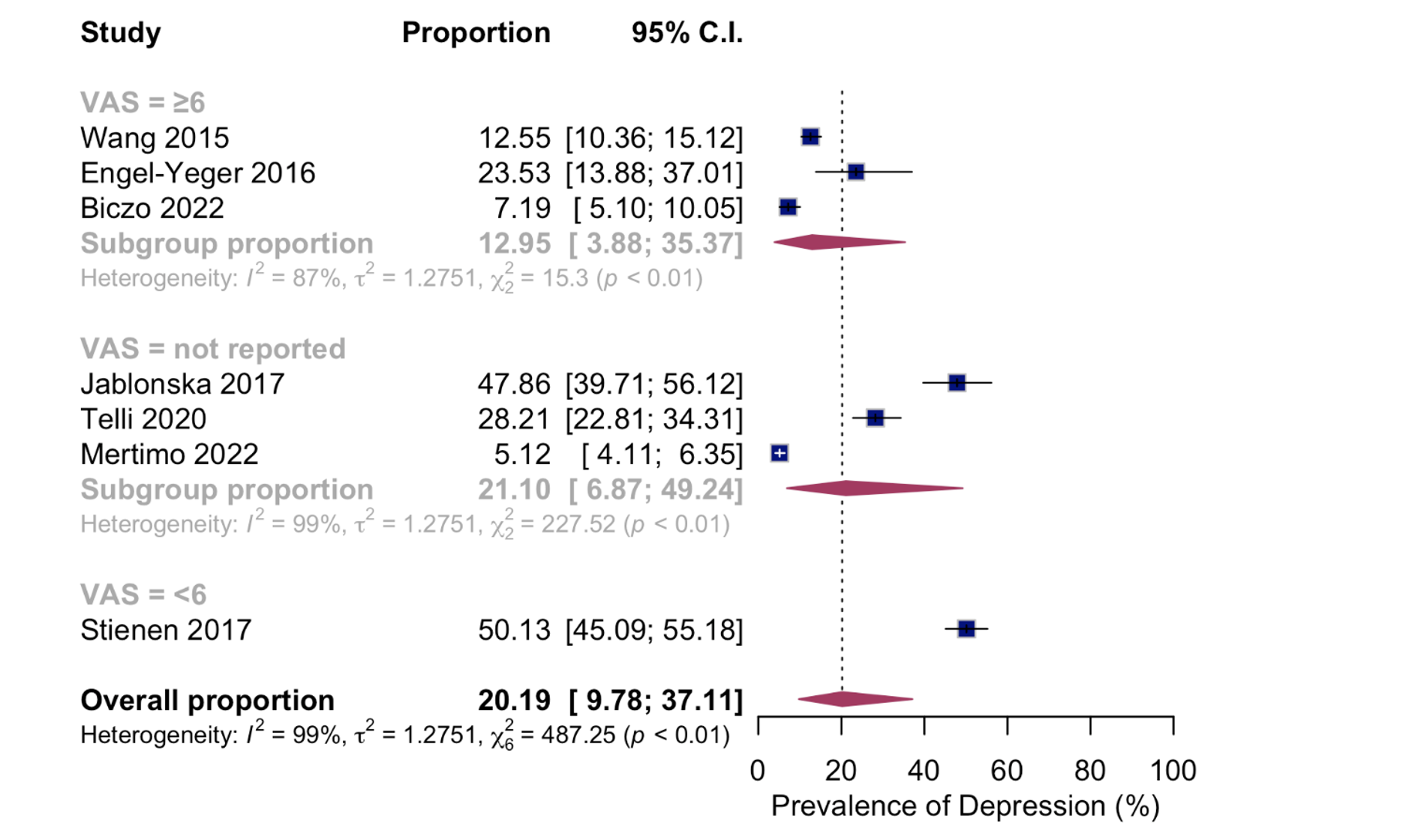

Supplement: S6 Fig — (TIF) [file pone.0322123.s007.tif]

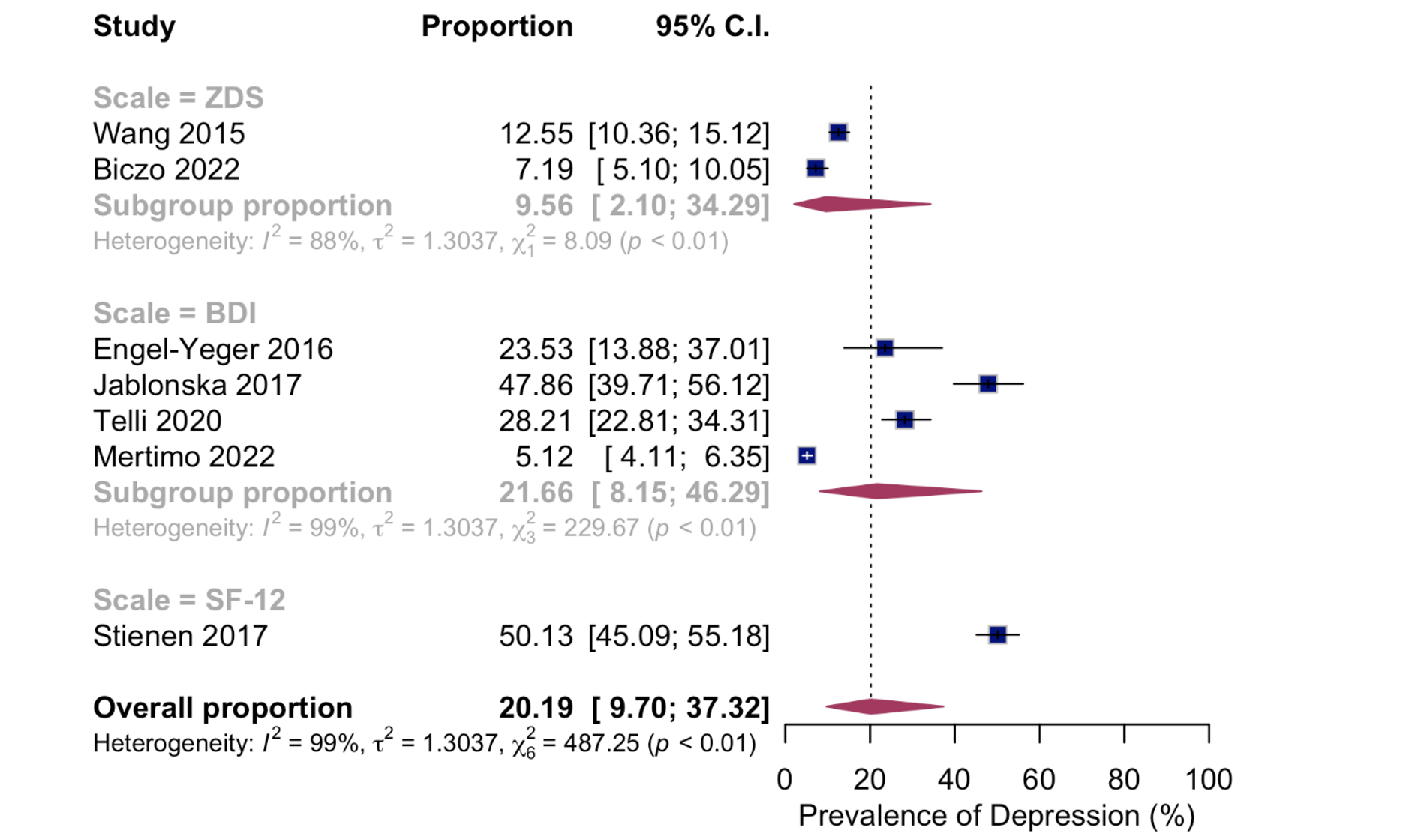

Supplement: S7 Fig — (TIF) [file pone.0322123.s008.tif]
