# Supplementary material for: The prevalence of depression in patients with lumbar degenerative disk disease: A systematic review and meta-analysis
Source: PLoS One. 2025 May 7;20(5):e0322123. doi: 10.1371/journal.pone.0322123 (PMC12057953; doi:10.1371/journal.pone.0322123)
Supplement: S2 File — (PDF) [file pone.0322123.s012.pdf]

National Institute for Health Research

University of York
Centre for Reviews and Dissemination

Systematic review

A list of fields that can be edited in an update can be found here

1. * Review title.

Give the title of the review in English

The prevalence of depression in patients with degenerative disk disease: A systematic review and meta-analysis

2. Original language title.

For reviews in languages other than English, give the title in the original language. This will be displayed with the English language title.

3. * Anticipated or actual start date.

Give the date the systematic review started or is expected to start.

07/01/2024

4. * Anticipated completion date.

Give the date by which the review is expected to be completed.

15/04/2024

5. * Stage of review at time of this submission.

This field uses answers to initial screening questions. It cannot be edited until after registration.

Tick the boxes to show which review tasks have been started and which have been completed.

Update this field each time any amendments are made to a published record.

The review has not yet started: No

Review stage	Started	Completed
Preliminary searches	Yes	No
Piloting of the study selection process	Yes	No
Formal screening of search results against eligibility criteria	No	No
Data extraction	No	No
Risk of bias (quality) assessment	No	No
Data analysis	No	No

Provide any other relevant information about the stage of the review here.

6. * Named contact.

The named contact is the guarantor for the accuracy of the information in the register record. This may be any member of the review team.

Indira Karibayeva

Email salutation (e.g. "Dr Smith" or "Joanne") for correspondence:

Dr. Karibayeva

7. * Named contact email.

Give the electronic email address of the named contact.

indira.karibayeva@gmail.com

8. Named contact address

Give the full institutional/organisational postal address for the named contact.

220 Lanier Dr, Apr 29, Statesboro, GA, 30458

9. Named contact phone number.

Give the telephone number for the named contact, including international dialling code.

+19124057997

10. * Organisational affiliation of the review.

Full title of the organisational affiliations for this review and website address if available. This field may be

completed as 'None' if the review is not affiliated to any organisation.

Georgia Southern University

Organisation web address:

https://www.georgiasouthern.edu/jphcoph/

11. * Review team members and their organisational affiliations.

Give the personal details and the organisational affiliations of each member of the review team. Affiliation refers to groups or organisations to which review team members belong. **NOTE: email and country now MUST be entered for each person, unless you are amending a published record.**

Dr Indira Karibayeva. Georgia Southern University
Dr Natalya Glushkova. Al-Farabi Kazakh National University

12. * Funding sources/sponsors.

Details of the individuals, organizations, groups, companies or other legal entities who have funded or sponsored the review.

no funding

Grant number(s)

State the funder, grant or award number and the date of award

13. * Conflicts of interest.

List actual or perceived conflicts of interest (financial or academic).

None

14. Collaborators.

Give the name and affiliation of any individuals or organisations who are working on the review but who are not listed as review team members. **NOTE: email and country must be completed for each person, unless you are amending a published record.**

15. * Review question.

State the review question(s) clearly and precisely. It may be appropriate to break very broad questions down into a series of related more specific questions. Questions may be framed or refined using PI(E)COS or similar where relevant.

What is the prevalence of depression among patients with degenerative disk disease (DDD)?

16. * Searches.

State the sources that will be searched (e.g. Medline). Give the search dates, and any restrictions (e.g. language or publication date). Do NOT enter the full search strategy (it may be provided as a link or

attachment below.)

We will search PubMed, Web of Science, ScienceDirect, Academic Search Complete and Google Scholar between January 2024 and March 2024. All searches are restricted to English language with no publication date restriction. Additional filters will be applied to include only research or peer review articles.

17. URL to search strategy.

Upload a file with your search strategy, or an example of a search strategy for a specific database, (including the keywords) in pdf or word format. In doing so you are consenting to the file being made publicly accessible. Or provide a URL or link to the strategy. Do NOT provide links to your search **results**.

https://www.crd.york.ac.uk/PROSPEROFILES/489684_STRATEGY_20240307.pdf

Alternatively, upload your search strategy to CRD in pdf format. Please note that by doing so you are consenting to the file being made publicly accessible.

Do not make this file publicly available until the review is complete

18. * Condition or domain being studied.

Give a short description of the disease, condition or healthcare domain being studied in your systematic review.

Degenerative disc disease (DDD) is characterized by the gradual breakdown of intervertebral discs in the spine, leading to symptoms such as chronic back pain and reduced spinal flexibility. Risk factors include aging, genetics, and lifestyle factors. Treatment options range from conservative measures like physical therapy to more invasive interventions such as surgery, with considerations for managing associated conditions like depression, which can often coexist with chronic pain.

19. * Participants/population.

Specify the participants or populations being studied in the review. The preferred format includes details of both inclusion and exclusion criteria.

The studies will be selected based on the following inclusion criteria: (a) studies reporting data on patients with degenerative disc disease (DDD); (b) studies reporting depression prevalence identified using a validated self-report questionnaire; (c) full-text publications in English in peer-reviewed journals. Exclusion criteria: (a) studies evaluating psychometric properties of instruments; (b) studies that did not specify the diagnosis of the included population; (c) studies with duplicative data; (d) studies reporting the number of patients with clinically diagnosed depression or receiving antidepressant treatment;

20. * Intervention(s), exposure(s).

Give full and clear descriptions or definitions of the interventions or the exposures to be reviewed. The preferred format includes details of both inclusion and exclusion criteria.

no intervention/exposure is targeted

21. * Comparator(s)/control.

Where relevant, give details of the alternatives against which the intervention/exposure will be compared (e.g. another intervention or a non-exposed control group). The preferred format includes details of both inclusion and exclusion criteria.

No control group is targeted

22. * Types of study to be included.

Give details of the study designs (e.g. RCT) that are eligible for inclusion in the review. The preferred format includes both inclusion and exclusion criteria. If there are no restrictions on the types of study, this should be stated.

Longitudinal and cross-sectional

23. Context.

Give summary details of the setting or other relevant characteristics, which help define the inclusion or exclusion criteria.

Only studies that specifically present information on the prevalence of depression based on the results of validated questionnaires will be included.

24. * Main outcome(s).

Give the pre-specified main (most important) outcomes of the review, including details of how the outcome is defined and measured and when these measurement are made, if these are part of the review inclusion criteria.

The main outcome of interest is the prevalence of depression among patients with degenerative disc disease (DDD), as determined by results from validated questionnaires administered either presurgery or during conservative treatment.

Measures of effect

Please specify the effect measure(s) for you main outcome(s) e.g. relative risks, odds ratios, risk difference, and/or 'number needed to treat.

Not applicable

25. * Additional outcome(s).

List the pre-specified additional outcomes of the review, with a similar level of detail to that required for main outcomes. Where there are no additional outcomes please state ‘None’ or ‘Not applicable’ as appropriate to the review

Not applicable

Measures of effect

Please specify the effect measure(s) for you additional outcome(s) e.g. relative risks, odds ratios, risk difference, and/or 'number needed to treat.

Not applicable

26. * Data extraction (selection and coding).

Describe how studies will be selected for inclusion. State what data will be extracted or obtained. State how this will be done and recorded.

According to the PRISMA guidelines, two of the authors will independently extract the data
information from the identified full-text articles using a standard data extraction form. The following of interest are: (1) the first author’s name, (2) publication year, (3) country, (4) study design, (5) sample size, (6) lesion location, (7) number of females, (8) number of smokers, (9) mean age, (10) type of work (physical, intellectual) (11) depression prevalence, (12) visual analogue scale (VAS) scores, (13) assessment method and criteria, (14) ODI scores. Any disagreement or conflict will be resolved by reaching a consensus through discussion.

27. * Risk of bias (quality) assessment.

State which characteristics of the studies will be assessed and/or any formal risk of bias/quality assessment tools that will be used.

The Critical Appraisal Skills Programme (CASP) Qualitative Research Checklist will be employed to evaluate the methodological quality of the included studies. This checklist comprises ten questions covering various aspects, such as the study's objectives, methodology, research design, recruitment approach, data collection methods, researcher-participant relationships, ethical considerations, data analysis, research findings, and overall value. Each criterion will be assessed with a rating of 'yes' when adequately described (scored as 1), 'no' when absent (scored as 0), and 'can't tell' when unclear or incomplete (scored as 0.5). The total scores will range from 0 to 10, with a score of at least 7 considered indicative of satisfactory quality.

28. * Strategy for data synthesis.

Describe the methods you plan to use to synthesise data. This **must not be generic text** but should be **specific to your review** and describe how the proposed approach will be applied to your data. If meta-analysis is planned, describe the models to be used, methods to explore statistical heterogeneity, and software package to be used.

The pooled mean depression prevalence with 95% confidence intervals (95% CI) will be calculated using random-effects model for meta-analysis in RevManager software. Heterogeneity across studies will be assessed using the I^2-statistic. Sensitivity analysis will be conducted to investigate which study significantly influences the pooled prevalence estimates, and a publication bias assessment will be conducted. Additionally, meta-regression analyses will be performed for age, gender, and baseline survey year.

29. * Analysis of subgroups or subsets.

State any planned investigation of ‘subgroups’. Be clear and specific about which type of study or participant will be included in each group or covariate investigated. State the planned analytic approach.

Subgroup analysis will be conducted to explore sources of heterogeneity, stratifying studies by study design, regions, lesion location, and assessment method.

30. * Type and method of review.

Select the type of review, review method and health area from the lists below.

Type of review

Cost effectiveness
No

Diagnostic
No

Epidemiologic
No

Individual patient data (IPD) meta-analysis
No

Intervention
No

Living systematic review
No

Meta-analysis
Yes

Methodology
No

Narrative synthesis
No

Network meta-analysis
No

Pre-clinical
No

Prevention
No

Prognostic
No

Prospective meta-analysis (PMA)
No

Review of reviews
No

Service delivery

No

Synthesis of qualitative studies

No

Systematic review

Yes

Other

No

Health area of the review

Alcohol/substance misuse/abuse

No

Blood and immune system

No

Cancer

No

Cardiovascular

No

Care of the elderly

No

Child health

No

Complementary therapies

No

COVID-19

No

Crime and justice

No

Dental

No

Digestive system

No

Ear, nose and throat

No

Education

No

Endocrine and metabolic disorders

No

Eye disorders

No

General interest

No

Genetics

No

Health inequalities/health equity

No

Infections and infestations

No

International development

No

Mental health and behavioural conditions

Yes

Musculoskeletal

Yes

Neurological

No

Nursing

No

Obstetrics and gynaecology

No

Oral health

No

Palliative care

No

Perioperative care

No

Physiotherapy

No

Pregnancy and childbirth

No

Public health (including social determinants of health)

No

Rehabilitation

No

Respiratory disorders

No

Service delivery

No

Skin disorders

No

Social care

No

Surgery

No

Tropical Medicine

No

Urological

No

Wounds, injuries and accidents

No

Violence and abuse

No

31. Language.

Select each language individually to add it to the list below, use the bin icon to remove any added in error.

English

There is not an English language summary

32. * Country.

Select the country in which the review is being carried out. For multi-national collaborations select all the countries involved.

United States of America

33. Other registration details.

Name any other organisation where the systematic review title or protocol is registered (e.g. Campbell, or The Joanna Briggs Institute) together with any unique identification number assigned by them. If extracted data will be stored and made available through a repository such as the Systematic Review Data Repository (SRDR), details and a link should be included here. If none, leave blank.

34. Reference and/or URL for published protocol.

If the protocol for this review is published provide details (authors, title and journal details, preferably in

Vancouver format)

Add web link to the published protocol.

Or, upload your published protocol here in pdf format. Note that the upload will be publicly accessible.

No I do not make this file publicly available until the review is complete

Please note that the information required in the PROSPERO registration form must be completed in full even if access to a protocol is given.

35. Dissemination plans.

Do you intend to publish the review on completion?

Yes

Give brief details of plans for communicating review findings.?

We plan to publish within the MDPI journal family.

36. Keywords.

Give words or phrases that best describe the review. Separate keywords with a semicolon or new line. Keywords help PROSPERO users find your review (keywords do not appear in the public record but are included in searches). Be as specific and precise as possible. Avoid acronyms and abbreviations unless these are in wide use.

Degenerative disk disease; Depression; Prevalence; Systematic review; Meta-analysis

37. Details of any existing review of the same topic by the same authors.

If you are registering an update of an existing review give details of the earlier versions and include a full bibliographic reference, if available.

Not applicable

38. * Current review status.

Update review status when the review is completed and when it is published.New registrations must be ongoing so this field is not editable for initial submission.

Please provide anticipated publication date

Review_Ongoing

39. Any additional information.

Provide any other information relevant to the registration of this review.

40. Details of final report/publication(s) or preprints if available.

Leave empty until publication details are available OR you have a link to a preprint (NOTE: this field is not editable for initial submission). List authors, title and journal details preferably in Vancouver format.

Give the link to the published review or preprint.
